# ViBrism DB: an interactive search and viewer platform for 2D/3D anatomical images of gene expression and co-expression networks

**DOI:** 10.1093/nar/gky951

**Published:** 2018-10-29

**Authors:** Masahiko Morita, Kazuro Shimokawa, Masaomi Nishimura, Sakiko Nakamura, Yuki Tsujimura, Satoko Takemoto, Takehiro Tawara, Hideo Yokota, Shuhei Wemler, Daisuke Miyamoto, Hidetoshi Ikeno, Akira Sato, Teiichi Furuichi, Norio Kobayashi, Yoshihiro Okumura, Yoko Yamaguchi, Yuko Okamura-Oho

**Affiliations:** 1 RIKEN Center for Advanced Photonics, Wako, Saitama 351-0198, Japan; 2 Tohoku Medical Megabank Organization, Tohoku University, Sendai, Miyagi 980-8575, Japan; 3 Wemler Software, Nerima, Tokyo 177-0042, Japan; 4 Graduate School of Engineering, The University of Tokyo, Meguro, Tokyo 153-0041, Japan; 5 School of Human Science and Environment, University of Hyogo, Himeji, Hyogo 670-0092, Japan; 6 Faculty of Science and Technology, Tokyo University of Science, Noda, Chiba 278-8510, Japan; 7 Head Office for Information Systems and Cybersecurity, RIKEN, Wako, Saitama 351-0198, Japan; 8 RIKEN Center for Brain Science, Wako, Saitama 351-0198, Japan; 9 Faculty of Human Life Science, Jissen Women's University, Hino, Tokyo 191-8510, Japan

## Abstract

Understanding anatomical structures and biological functions based on gene expression is critical in a systemic approach to address the complexity of the mammalian brain, where >25 000 genes are expressed in a precise manner. Co-expressed genes are thought to regulate cell type- or region-specific brain functions. Thus, well-designed data acquisition and visualization systems for profiling combinatorial gene expression in relation to anatomical structures are crucial. To this purpose, using our techniques of microtomy-based gene expression measurements and WebGL-based visualization programs, we mapped spatial expression densities of genome-wide transcripts to the 3D coordinates of mouse brains at four post-natal stages, and built a database, ViBrism DB (http://vibrism.neuroinf.jp/). With the DB platform, users can access a total of 172 022 expression maps of transcripts, including coding, non-coding and lncRNAs in the whole context of 3D magnetic resonance (MR) images. Co-expression of transcripts is represented in the image space and in topological network graphs. In situ hybridization images and anatomical area maps are browsable in the same space of 3D expression maps using a new browser-based 2D/3D viewer, BAH viewer. Created images are shareable using URLs, including scene-setting parameters. The DB has multiple links and is expandable by community activity.

## INTRODUCTION

Associated information involving anatomical structures and biological functions based on gene expression profiles is critical in systemically approaching complex structures, such as the mammalian brain, where >25 000 genes are expressed (1). Indeed, non-random combinations of expressed genes are required for developmentally deliberate formation of functional regions. The activation of specific subsets of genes, referred to as co-expressed genes, is thought to regulate cell type- or region-specific brain functions (2). Thus, well-designed data acquisition and visualization systems for profiling combinatorial gene expression in relation to anatomical structures are useful for the research community in understanding brain complexity.

For this purpose, using our invented microtomy-based gene expression measurement technique, Transcriptome Tomography, we mapped spatial expression densities of genome-wide transcripts on the 3D MR image space of mouse brains at four post-natal stages, and built a database, ViBrism DB (http://vibrism.neuroinf.jp/).

In the ViBrism DB viewer system, users can see coding and non-coding transcripts and lncRNAs expressed in particular areas in the whole anatomical context of the 3D MR image space. Users are informed about co-expression of transcripts in the image space and in topological network graphs. *In situ* hybridization (ISH) images at a cell-level resolution and anatomical area maps are browsable in the same space of 3D expression maps using a Brain Atlas Hackathon (BAH) viewer. Created images on the browser are sharable by employing URLs, showing scene-setting parameters. The DB has multiple links to and from external DBs and is expandable by ongoing community activity.

## MATERIALS AND METHODS

### Ethics statement

All procedures involving animals and their care were performed according to the RIKEN Regulations for Animal Experiments (approval IDs: H25-2-106(2) and H25-2-004(1)).

### System design and implementation

**Figure 1.**
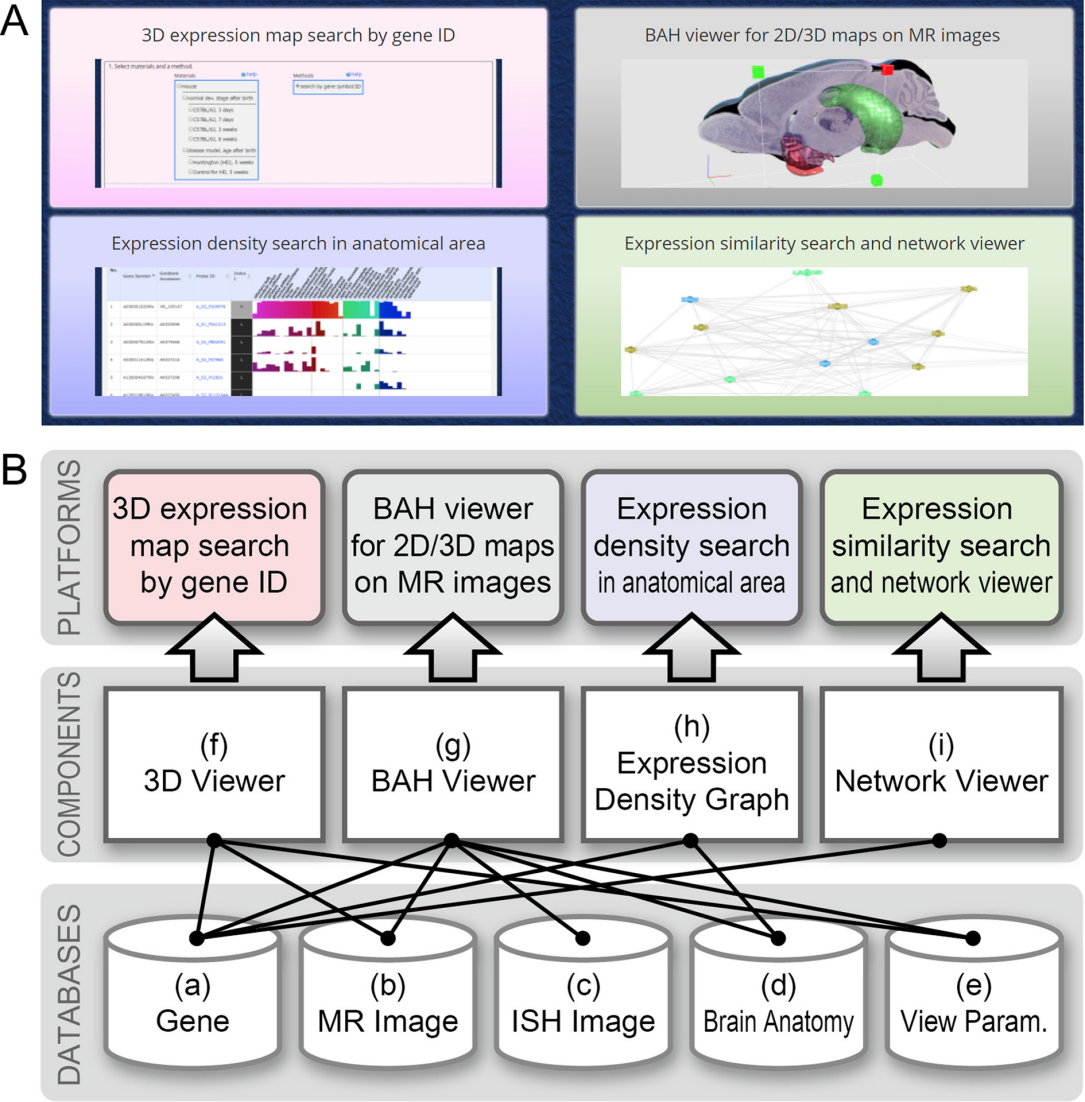
Platform services and the system component. (**A**) Icons at the top page of the ViBrism DB, which represent services of the four platforms. (**B**) System components and databases for platform services. Data details are characterized in the methods section. (a) The gene database stores 3D expression map files, the gene ID data, the variables data, the Pearson correlation coefficient data, and the gene expression density data in the anatomical areas. (b) The MR image database manages MR images of each virtual brain. (c) The ISH image database currently manages ISH images in the virtual brain at the 8-week stage. (d) The brain anatomy database stores anatomical area maps for visualization in the BAH viewer. (e) The view parameter database manages scene-setting parameters for 3D and BAH viewers. (f) The 3D viewer, which has been developed using WebGL, and matrix operation API glMatrix (http://glmatrix.net/) provides a 3D visualization function of expression maps on MR images for the platform of ‘3D expression map search by gene ID’. (g) The BAH viewer is an extension of the 3D viewer for ISH 2D image visualization. This component has been enabled in the platform of ‘BAH viewer for 2D/3D maps on MR images’. (h) The expression density graph displays volume ratios of expression to each anatomical area volume. This component is used in the platform of ‘Expression density search in anatomical area’. (i) The network viewer has been developed using a graph visualization library in arbor (http://arborjs.org) to depict stochastic network graphs for the platform ‘Expression similarity search and network viewer’.

The current version of ViBrism DB has been developed mainly using MySQL 5.7.22 (https://www.mysql.com/) for relational database management system (RDBMS), Apache httpd 2.2.15 (https://httpd.apache.org/) for HTTP server, and PHP 5.6.36 (http://www.php.net/) for server-side scripting language. The web user-interface has been written using PHP, JavaScript, CSS and HTML5, and deploys Bootstrap framework (http://getbootstrap.com/) and JQuery library (https://jquery.com/). We recommend using a modern web browser running HTML5 and a Web Graphics Library (WebGL), such as Firefox (preferred), Google Chrome or Safari, to achieve the best display effect. To provide the services across four platforms, the system consists of components and databases as illustrated in Figure 1.

### Materials

Virtual brains at four post-natal age stages were made from real brains of C57BL/6J mice at 3 days, 7 days, 3 weeks and 8 weeks after birth (Table 1). Comprehensive gene expression densities of the whole brains were measured and 3D mapped with the TT method (3), as briefly described below. MR images and anatomical area maps of the 8-week-old brain were adopted from the WHS standard mouse image database (4); the images are downloadable from https://www.nitrc.org/projects/incfwhsmouse, and were used as the standard coordinates for the 8-week datasets. MR images at the other age stages were obtained by nuclear magnetic resonance (Bruker Bio Spin, AVANCE 400WB) at 9.4 Tesla at a spatial resolution of 100 μm and used as the coordinate space for each stage. ISH images, in which transcripts were histologically stained in 2D slices, were adopted from the BrainTx database (a mouse brain gene expression database http://www.cdtdb.neuroinf.jp/) (5), and transformed to the WHS coordinates system as described below.

**Table 1.** Datasets and analysis platforms available in ViBrism DB

Virtual brain age after birth	3 days	7 days	3 weeks	8 weeks
Mouse strain	C57BL/6J	C57BL/6J	C57BL/6J	C57BL/6J
**Gene expression datasets created with Transcriptome Tomography (TT)**				
Number of materials used	3 mice	3 mice	3 mice	6 mice
Fraction thickness (resolution)	500 μm	1000 μm	1000 μm	∼1000 μm^a^
Fraction number (data points)	34	22	28	61
Microarray system	SurePrint G3 Mouse GE 028005			Whole Mouse Genome 012694
Types of detectable transcripts	coding, non-coding, lnc (27,122 Entrez Genes and 4.578 lncRNAs)			coding, non-coding
Number of transcripts detected	43 199	47 544	44 721	36 558
GEO ID	GSE118176	GSE118177	GSE118178	GSE36408
**Other datasets**				
MRI	own made T1, T2W	own made T1, T2W	own made T1, T2W	WHS standard T1, T2^a^, T2W
Anatomical area	—	—	—	WHS 35 areas
ISH image	—	—	—	2810 images
**Analysis platforms**				
1. search by gene ID	Available	Available	Available	Available
2. map viewer	3D map viewer	3D map viewer	3D map viewer	2D/3D map viewer (BAH viewer)
2D thumbnail	Available	Available	Available	Available
3. SET search and network viewer	Available	Available	Available	Available
4. anatomical area search	—	—	—	Available

^a^The fraction size is 1000 μm in depth and duplicated in each direction. Consequently, map resolution is smaller than 1,000 μm.

### Transcriptome tomography (TT)

A schema of the method is provided in Supplementary Figure S1. Two types of data were obtained from brain fractions via a sequential cross-sectioning (i) gene expression densities in the fractions and (ii) block-face images of cross-sectioning planes. The cross-sectioning series was performed throughout the whole brain in one of the orthogonal directions, so that, at least three brains were required. The data were used for the 3D reconstruction of gene expression maps in the MR image coordinates with tomographic methods to create a virtual brain. Expression data of the 8-week old brain, as previously reported (3), were re-mapped to the standard WHS MR images, otherwise all maps were newly produced.

### Fraction data analyses

The gene expression densities in the fractions were measured as the intensity data on microarray probes that specifically bound to transcripts. The intensity data were per-chip normalized (hereafter referred to as fraction data). The probes are sufficient to detect most coding and non-coding transcripts. lncRNAs were also detected in the fractions of the 3-day, 7-day and 3-week brains. The microarray data were deposited with the NCBI Gene Expression Omnibus database (6) and are accessible through GEO Series accession number (Table 1).

### Variables of gene expression density data for each transcript

Four sets of variables, I, V, D and K were calculated from the log-transformed fraction data for each transcript in each virtual brain and stored in the gene database (Figure 1B). Variable I and V represent the intensity medians and the expression variance, respectively, as previously described (3). At 3-day, 7-day and 3-week stages, a coefficient of variation is used for variable V. Variables I and V are indicated with five and three grades, respectively, with the threshold shown in the instruction tab https://vibrism.neuroinf.jp/instruction.html.

Variable D represents the detection rate. Transcripts detected in at least one fraction of a sectioning series in all the series were indicated with Y.

Variable K represents the number of similarly expressed probes to a target probe (SET) including itself. The Pearson Correlation coefficient (*r*) of the fraction data between two probes was calculated and used as a correlation measure for expression similarity (7). The default threshold of r was set at 0.85 and variable K minus one, which means the number of SET excluding itself, is shown in the columns of SET.

### Network graphs

The r value matrixes at thresholds of *r* > 0.7, 0.75, 0.8, 0.85, 0.90, and <–0.7, –0.75, –0.8, –0.85, –0.90 between all transcripts at each post-natal stage were calculated and stored in the gene database (Figure 1B). Stochastic graphs of co-expression networks at the user specified threshold were interactively depicted in browsers with an open source program of the Barnes-Hut algorithm provided from a graph visualization library, arbor.js https://github.com/samizdatco/arbor.

### Anatomical map analysis

High expression areas of transcripts were defined using an 80% cut-off filter (Supplementary Figure S1). An expression level of the transcript in a WHS anatomical area was defined as $\frac{{{\rm{N\ }}( {{\rm{Ve\ }} \cap {\rm{\ Va}}} )}}{{{\rm{N\ }}( {{\rm{Va}}} )}},$ in which Ve is the voxel area of transcript ‘e’ high expression, Va is the voxel area of the anatomical area ‘a’; and *N* is the voxel number of the areas in parentheses. The calculated results of all transcripts in 35 anatomical areas of the 8-week brain was stored in the database and are shown as bar graphs in the platform of ‘Expression density search in anatomical areas’ (Figure 1A).

### ISH images of genes transformed to the WHS standard coordinates

Para-sagittal section images of brains stained with ISH methods were subjected to registration to the WHS standard coordinate space. The ISH images were linearly transformed into each of a series of para-sagittal MR image slices, and the similarity metric value (δ) of the two images were calculated. The para-sagittal MR image with the smallest δ was selected as the best-fit slice and the ISH image was overlaid on that slice surface in the coordinate space. In total, 2810 high resolution digital images were transformed (D. Miyamoto *et al., bioRxiv Prepr*., https://doi.org/10.1101/386086). Transformed ISH images, along with the best-fit slice numbers, were archived in the database (Figure 1B). Images were searchable and visualized in the ‘BAH viewer for 2D/3D maps on MR images’.

### 3D viewer and BAH 2D/3D viewer

The 3D viewer was originally invented as a desktop program to visualize a virtual surface image of a specimen (8). Volume rendering is popular for visualizing 3D volume images, but it requires considerable computational resources and an advanced graphics processing unit (GPU). Our basic approach to visualize sectioned planes of volume images by using surface rendering techniques, not volume rendering, is shown in Supplementary Figure S2. Our WebGL-based method requires less computational resources and GPU than volume rendering, so that it is usable by a web application for real-time interaction.

### Data sharing by way of URLs showing scene-setting parameters of the viewer

The 3D and BAH viewers have a unique system to save a current state of the camera viewing and object information as a URL containing parameter information as follows: a camera position; expression map IDs and anatomical area map IDs; their surface positions, colors and textures; a MR image ID; an ISH image ID and the slice position: and a sectioning position with a plane, a cube or a sphere. Users can temporally store scene-setting parameters of a current image in the view parameters database as a URL (Figure 1B) and send them to anyone. The image will be resumed by pasting the URL into a browser.

### Data downloadable from the platform

Co-expression network link tables in a CSV or GML format, which are compatible with open source software applications, such as Cytoscape, for further network analysis on desktop computers, are downloadable. Zipped files labeled with map-file IDs containing 3D expression map files are downloadable. These files are not required for the browser-based rendering here we introduced.

### Data accessibility from and to external databases

In order to promote open data publication and data integration on the web, the ViBrism dataset was converted into Resource Description Framework (RDF) as linked open data (LOD). It is accessible from RIKEN MetaDatabase http://metadb.riken.jp, which includes various RIKEN’s research resultant datasets and related public datasets of life sciences, having 160 databases and 30 ontologies as of August 2018 (9). The RDFised ViBrism database http://metadb.riken.jp/db/ViBrism contains ∼3 million RDF triples with 19 classes and are accessible by both human-friendly RIKEN MetaDatabase viewer and SPARQL queries for programs.

Information regarding gene ontology and expression maps in embryonic stages can be reached through links to BrainTx and EMAP/EMAGE (a mouse embryo gene expression database, https://www.emouseatlas.org/emap/home.html) (10), respectively.

### Instructions, help and notes

Quick instruction manuals for the four platforms in PDF format are downloadable from the ‘Instruction’ tab at the top page of the DB. Small ‘help’ icons are seen in search processes throughout the DB to introduce instruction sections for help. In downloading network tables, file sizes are limited, as described in the note.

## PLATFORM FUNCTIONALITY AND USE

### 3D expression map search by gene ID

**Figure 2.**
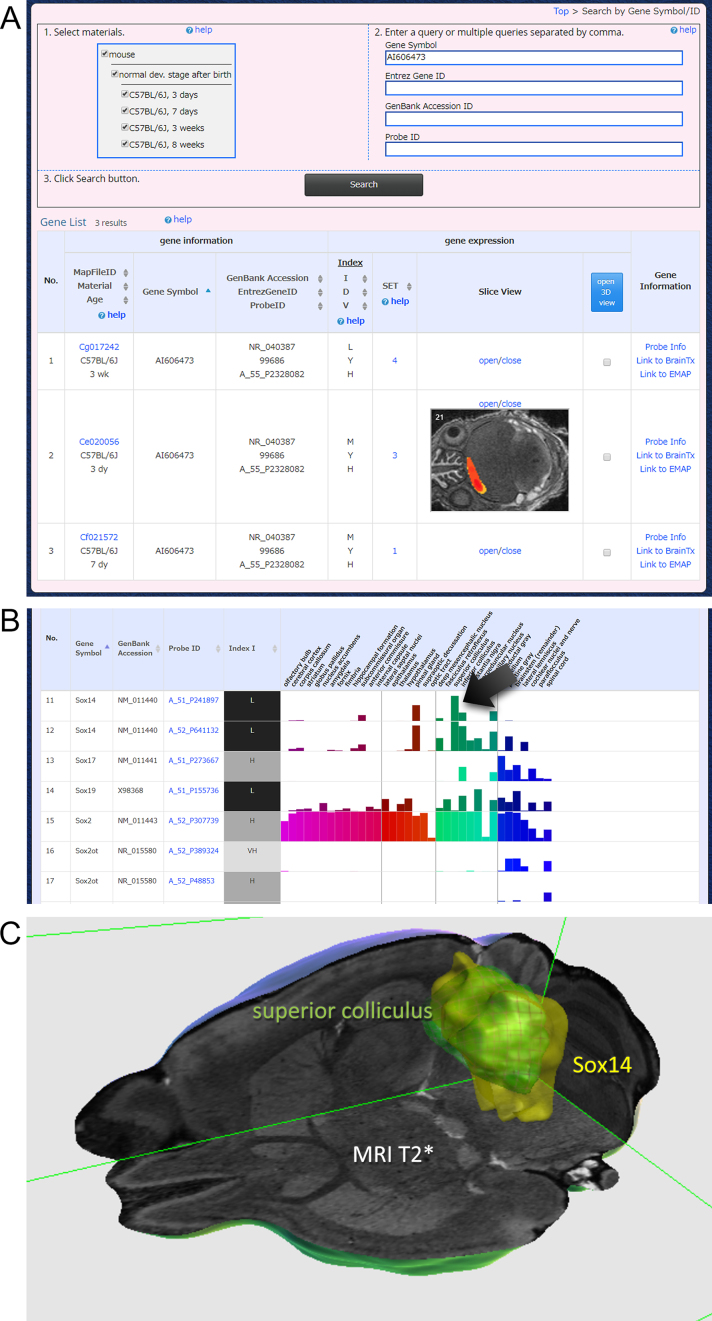
Search by gene IDs and results. (**A**) An example view of searching for genes of interest by gene IDs. Users are expected to follow instructions 1, 2, and 3, and then, find search results. A gene of interest, for instance, AI606473 is a non-coding transcript of unknown function. The result shows the gene is expressed at three post-natal stages in low (L) or medium (M) expression intensities (in the column of variable index I) with high (H) expression variance (variable index V) and has 1 to 4 similarly expressed genes (in the SET column). A 2D thumbnail at the 3-day stage is shown. 3D maps will be shown by checking square buttons and clicking the open 3D view icon. Probe information and external links are shown in the right column. (**B**) A search result for sox gene families using the gene symbol ‘sox’ in the platform of ‘expression density search in anatomical area’. A total of 22 transcripts were searched. The results are sorted by gene symbol, and a part of the results is shown. Expression levels of transcripts in the 35 anatomical areas are shown as bar graphs. Colors in bars correspond to the default color code of anatomical areas in the BAH viewer. (**C**) Click the bar (with an arrow), then BAH viewer showing the expression map and the anatomical area will open. An example view of sox14 gene expression map (yellow), superior colliculus (green with grids) on the T2* MR image cut with a cube is shown (the scene-setting parameter: https://vibrism.neuroinf.jp/setsearch/3d/view/Cx1/7c930528725581f005dc5dbea7eec8a3).

This platform is a gateway to other visualization platforms of ViBrism DB. A total of 172 022 maps of transcripts, including coding, non-coding and lncRNAs at the four post-natal stages, are searchable (Figure 2A). Users can search transcripts by gene symbols (examples: abcd, ef, fox, sox), Entrez gene IDs (99 686, 16 875, 20 669), GenBank accession IDs (NM_029002, NM_183248, XM_006499301) and Agilent microarray probe IDs (A_30_P01017714, A_30_P01030305). Comma-separated multiple queries are acceptable. Post-natal ages can be specified. Then, users will find the search result table showing IDs of selected genes and their variables. Thumbnails of expression maps in 2D can be seen, and 3D maps can be selected to browse on the 3D viewer. Users will proceed to similarly expressed gene (SET) search by clicking the number in the SET column. Microarray probe information, including lncRNAs, is present in the right column. External links are available.

### Expression density search in anatomical areas

This platform is available for the dataset of the 8-week age stage. Genes of interest are searchable and expression levels of the genes in the WHS anatomical areas are visualized in bar graphs (Figure 2B). By clicking a bar, the anatomical area and the expression map are shown in the BAH viewer (Figure 2C).

### BAH viewer for 2D/3D maps on MR images

**Figure 3.**
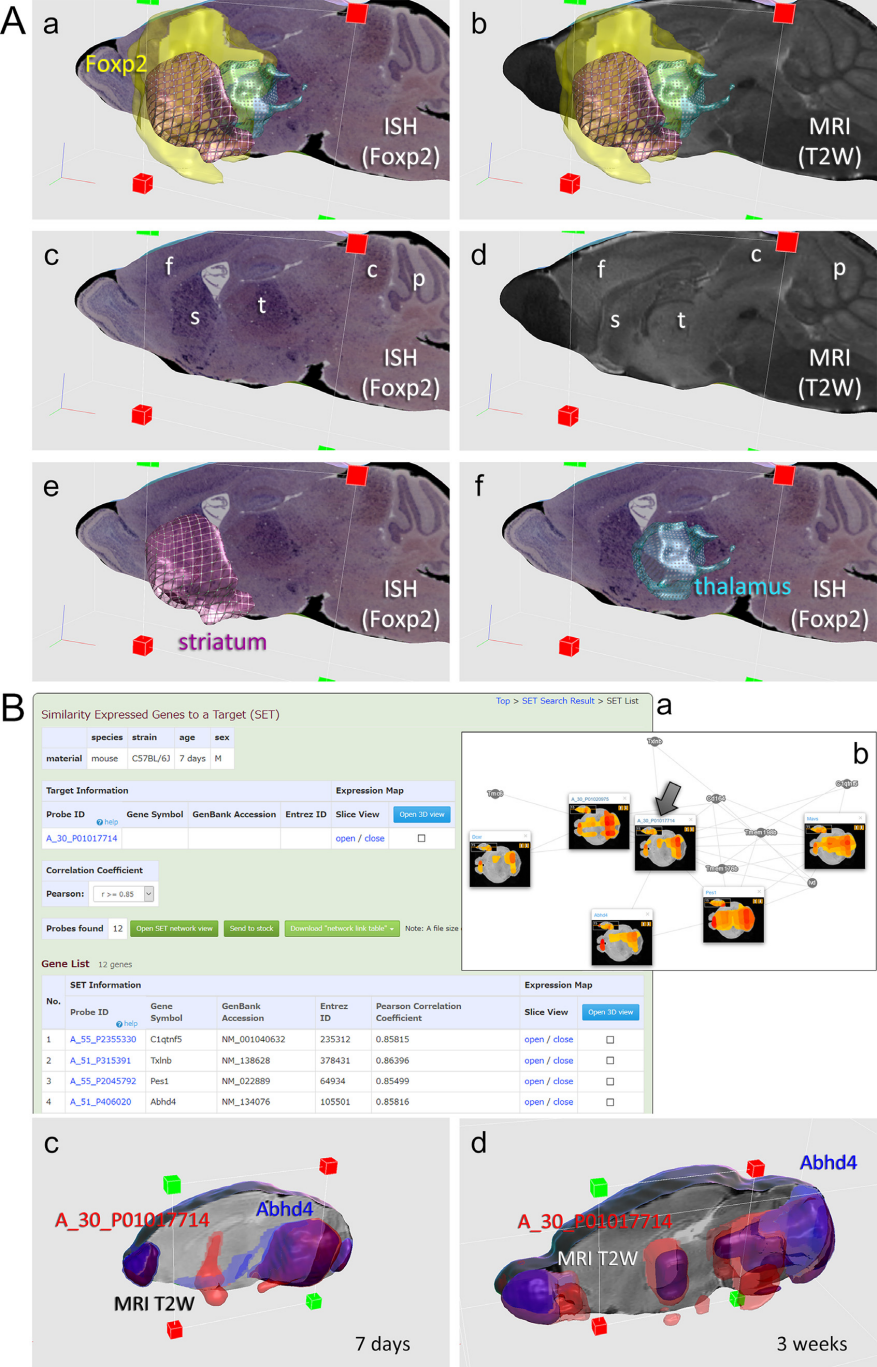
Example results in anatomical BAH viewer and topological network viewer. (**A**) Foxp2 is a gene for a transcription factor, which functions in brain development. In the BAH viewer at 8 weeks post-natal stage, (**a**) Foxp2 3D expression map (yellow) and the ISH image on the best-fit slice of the MR image are shown along with anatomical area maps of the striatum (purple) and the thalamus (light blue), (**b**) the 3D map and the anatomical area maps are shown in the same slice of the MR image, (**c**) and (**d**) the ISH image and the MR image comparable in the same section, and (**e**) and (**f**) the ISH image and the anatomical map. These images show that Foxp2 gene is still expressed in the 8-weeks matured brain, mostly in the forebrain, including the frontal cortex (f), the striatum (s) and the thalamus (t), and, in some mounts, presents in the colliculus and the Purkinje cell layers (the scene-setting parameter: https://vibrism.neuroinf.jp/setsearch/3d/view/Cx1/4ae7e07297a029ce7229f1808d32f8ca). (**B**) A_30_P01017714 is a lncRNA of unknown function. (**a**) SET search results at 7 days of post-natal stage are shown: 12 genes are present in the SET and several are shown. Buttons for open SET network graph, data stock, link table download, and open 3D view are shown. (**b**) An example of stochastic network graphs of SET of this gene (with an arrow) at 7 day post-natal stage. 2D thumbnails are shown. After data stock, calculating intersection of SET at 3 day, 7 day and 3 week stages in the platform, A_30_P01017714 is found to be co-expressed with Abhd4 at 7 days and 3 weeks. (**c**) and (**d**) Areas of co-expression are shown in the 3D viewer (the scene-setting parameter: at the 7-day stage: https://vibrism.neuroinf.jp/setsearch/3d/view/Cf0/27a5e2195222b578194ca22f15db1d5b and at the 3-week stage: https://vibrism.neuroinf.jp/setsearch/3d/view/Cg0/496aed8e85ed1bfee8eb62b2cd1d416b).

The BAH viewer enables users to interactively manipulate multiple 3D expression maps, 3D anatomical area maps and 2D ISH images in the MR image coordinates in a web browser (Figure 3A). Users first select maps of interest in the ‘3D expression map search by gene ID’ platform, then access this platform by clicking the 3D view button. For the brain at the 8-week stage, alternatively, users can start searching 2810 ISH images and 36 558 3D expression maps directly in this platform or start searching from the ‘Expression density search in anatomical area’ platform. ISH images and anatomical area maps are currently available only for the 8-week stage, such that the viewer system for the other stages is called 3D viewer: object manipulation in the browser are almost the same in the two viewers. Objects are fully rotated by dragging and virtually dissected with an arbitrary plane, a cube or a sphere. Users can share created images by saving the URLs of scene- setting parameters and sending them in some way. Images will be resumed by pasting the URLs into a browser.

### Expression similarity search and network viewer

In this platform, first, users print gene IDs of interest, which we call a target gene, and select a threshold of r for the SET search. On pressing the search button, users will find a list of candidate genes of interest and their SET number, which is the variable V, as described above. By clicking the SET number, users will proceed to browse the gene list of the SET member. In this results page, users can proceed to SET network viewer or 3D viewer. Alternatively, multiple results can be temporary stored for calculation of sum or intersection of the SET members. Next, the results are visualized with the SET network viewer as stochastic graphs of similar expression. Network link tables are downloadable. The 3D viewer is available to show the anatomical areas of co-expression (Figure 3B).

### Example analysis

Analysis of non-coding transcript AI606473 is shown in Figure 2A. Further SET analysis indicates it is co-expressed with Lhx8 and Isl1 (scene-setting parameter: https://vibrism.neuroinf.jp/setsearch/3d/view/Cg0/f3fce27b9a8576c26db0c65492671f39). Sox gene families are searched in Figure 2B and C. 3D expression maps and 2D ISH images of Foxp2 are visualized in Figure 3A. A_30_P01017714 is a lncRNA of unknown function. Search results (Figure 3B) indicates that this transcript is expressed mostly in the olfactory bulb, thalamus, midbrain and the cerebellum. SET analysis reveals its co-expressed with Abhd4, a major lipase for N-acyl phospholipid in the brain (11), in the 7-day and 3-week brains. This suggests some function of this lncRNA in lipid metabolism regulation in these areas during post-natal neural maturation.

## DISCUSSION AND FUTURE PLAN

ViBrism DB is an exclusive platform that contains original datasets of 3D gene expression maps and co-expression networks created with TT methods, and which allows web-based visualization of the datasets in new viewers. The DB platform enables researchers to find comprehensive gene expression density patterns in spatio-temporal diversity without restriction of materials to a single cell type by cell sorting or to selected areas by microdissection, but, rather, in the whole anatomical context of MR images. Now totaling 172 022 expression maps of the four brain datasets, along with MR images, the anatomical area maps and 2810 ISH images are freely available without requirement of any login or registration. There is no password required. Gene data is accessible from RIKEN MetaDatabase. Moreover, they have links to those in EMAP/EMAGE (Edinburgh, UK) and BrainTx (RIKEN, Japan). Those data together are contributing to community activities in molecular biology, developmental biology and genetics.

A widespread resource that comprehensively maps gene expression in the mouse brain is the Allen Mouse Brain Atlas (2004) and Allen Developing Mouse Brain Atlas (2008) (1,12), in which 3D gene expression maps of brains in adult and developing stages are reconstructed from ISH 2D images of very many brains, so that expression similarity can be searched in 4.5K selected genes with local expression. GENSAT provides precise gene expression 2D images using a sophisticated transgenic and staining technique of each gene (13). However, endogenous gene expression could not be quantified. Our mapping system is automated, is 3D-oriented and requires only 3–6 brains to profile expression maps. Endogenous transcripts are measured using microarray and the data are normalized. Consequently, our database is very powerful in expression similarity searches of 40K transcripts in the whole brain. Furthermore, Search results can be visualized topologically in the network viewer, along with 2D thumbnails of expression, and co-expression areas are shown anatomically on the corresponding MR images with the 3D viewer.

The WebGL-based visualization system is original and, hopefully, stress-free for interactive rendering of multiple 2D/3D images through web accessing. It provides exclusive views: ISH slice images can be seen in the 3D coordinates, together with expression maps, MR images and anatomical area maps.

The TT method is available for any researcher under their experimental conditions, and ViBrism DB is expandable for them. We will increase the number of virtual brains, including disease model mice. Moreover, 3D and 2D maps created by other researchers would be registered.

Since 2014, ViBrism DB has been hosted by RIKEN CBS (RIKEN BSI reorganized) as one of neuroinformatics platforms at the Japan Node of International Neuroinformatics Coordinating Facility (INCF) https://www.neuroinf.jp/program/platforms.html. Each platform has been providing a variety of information and contributions for the neuroscience community since 2005. ViBrism DB, in particular, has contributed to molecular genetics by inventing original methods of TT and launching the database in 2012 (3) and by offering co-expression analysis in 2014 (7): the methodology was referred to in a review issue of new molecular technology in 2015 (14).

Compared to the previous version, the new ViBrism DB introduced here is improved as follows: the number of virtual brains has been increased, images with different modalities (3D maps and 2D ISH images) are integrated, a new 3D viewer/BAH viewer is launched to interactively browse the images and to share images, and an advanced network viewer has been developed, in which network sum and intersects can be calculated. Here, for the first time, we have fully described ViBrism DB structures and functions for, hopefully, contributing to the broader science community.

## DATA AVAILABILITY

The microarray data at 3 days, 7 days and 3 weeks of post-natal stages have deposited with the NCBI Gene Expression Omnibus database (6) and are accessible through GEO Series accession numbers GSE118176, GSE118177 and GSE118178. The microarray data at the 8-week stage was previously deposited under accession number GSE36408.

## Supplementary Material

Supplementary Data
