# Supplementary material for: ViBrism DB: an interactive search and viewer platform for 2D/3D anatomical images of gene expression and co-expression networks
Source: Nucleic Acids Res. 2018 Oct 29;47(Database issue):D859–66. doi: 10.1093/nar/gky951 (PMC6324046; doi:10.1093/nar/gky951)
Supplement: Supplementary Data [file gky951_supplemental_files.zip › Supplementary_Figure_S1_Vibrism_0814.pdf]

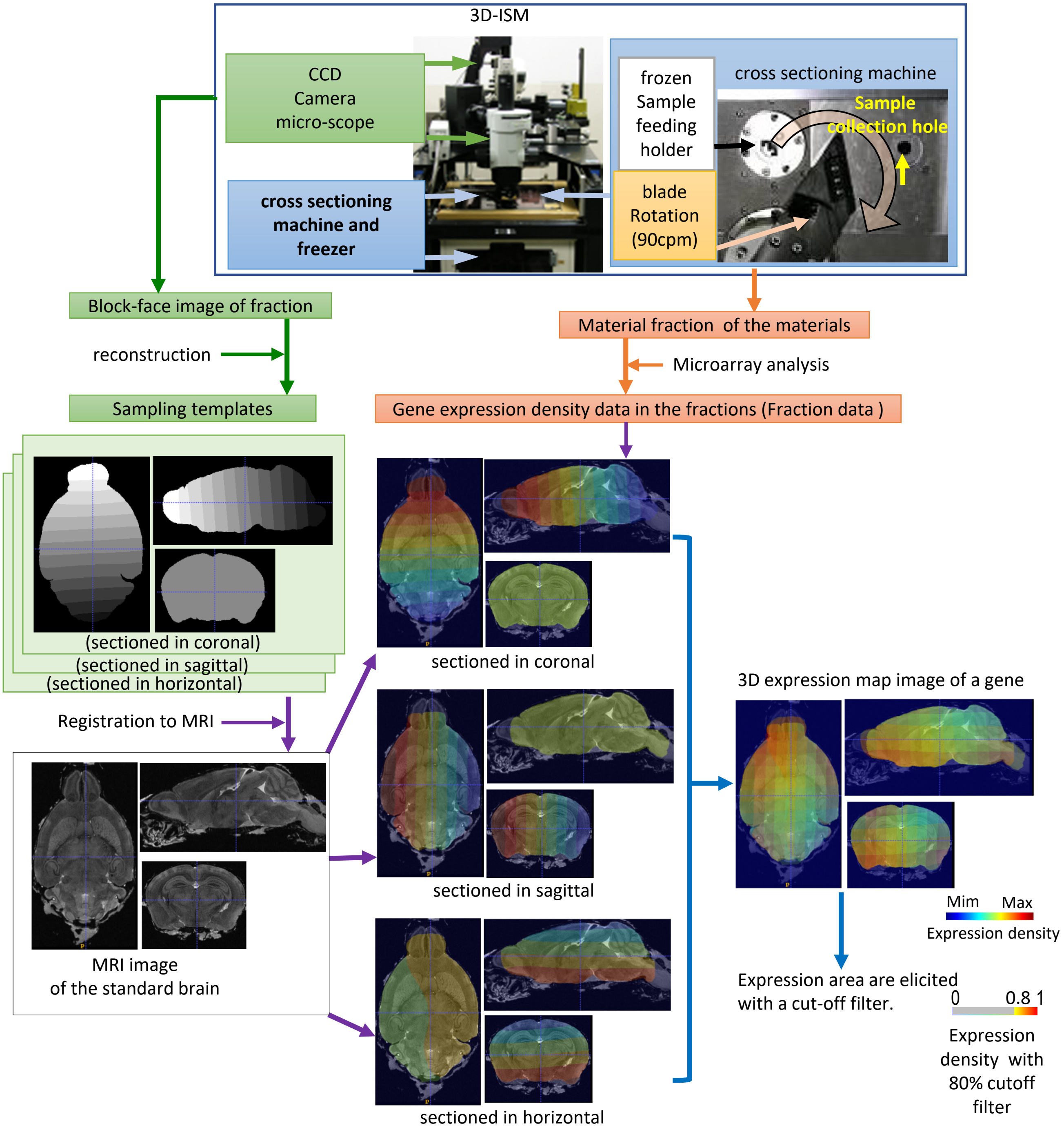

Supplementary Figure S1. Transcriptome Tomography Two types of data were obtained from a series of brain fractions via sequential cross-sectioning in multiple batches of 500 -1,000 µm (5 µm x 100-200 sections per batch)-thick fractions: 1) gene expression densities in the fractions measured by microarray (fraction data, colored in orange) and 2) block-face images of the 5 µm thick cross-sectioning planes (in green). This cross-sectioning series was performed throughout the whole brain in one of the orthogonal directions. At least three brains were required for the orthogonal sectioning series. The block-face images were reconstructed to create sampling templates, which showed fraction images in the thee brains. Compared to the previous report (3), we improved our mapping methods: we transformed the template images to the MR image of the standard brain, not to one template, using 3D non-rigid transformation protocols of ANTs in the ITK library. The fraction data were assigned to the voxels of the fraction images (indicated with purple arrows). A 3D expression map image of each gene were reconstructed by averaging fraction data in the voxels and visualized with pseudo colors (with blue arrows).
