# Supplementary material for: ViBrism DB: an interactive search and viewer platform for 2D/3D anatomical images of gene expression and co-expression networks
Source: Nucleic Acids Res. 2018 Oct 29;47(Database issue):D859–66. doi: 10.1093/nar/gky951 (PMC6324046; doi:10.1093/nar/gky951)
Supplement: Supplementary Data [file gky951_supplemental_files.zip › Supplementary_Figure_S2_ViBrism_0815.pdf]

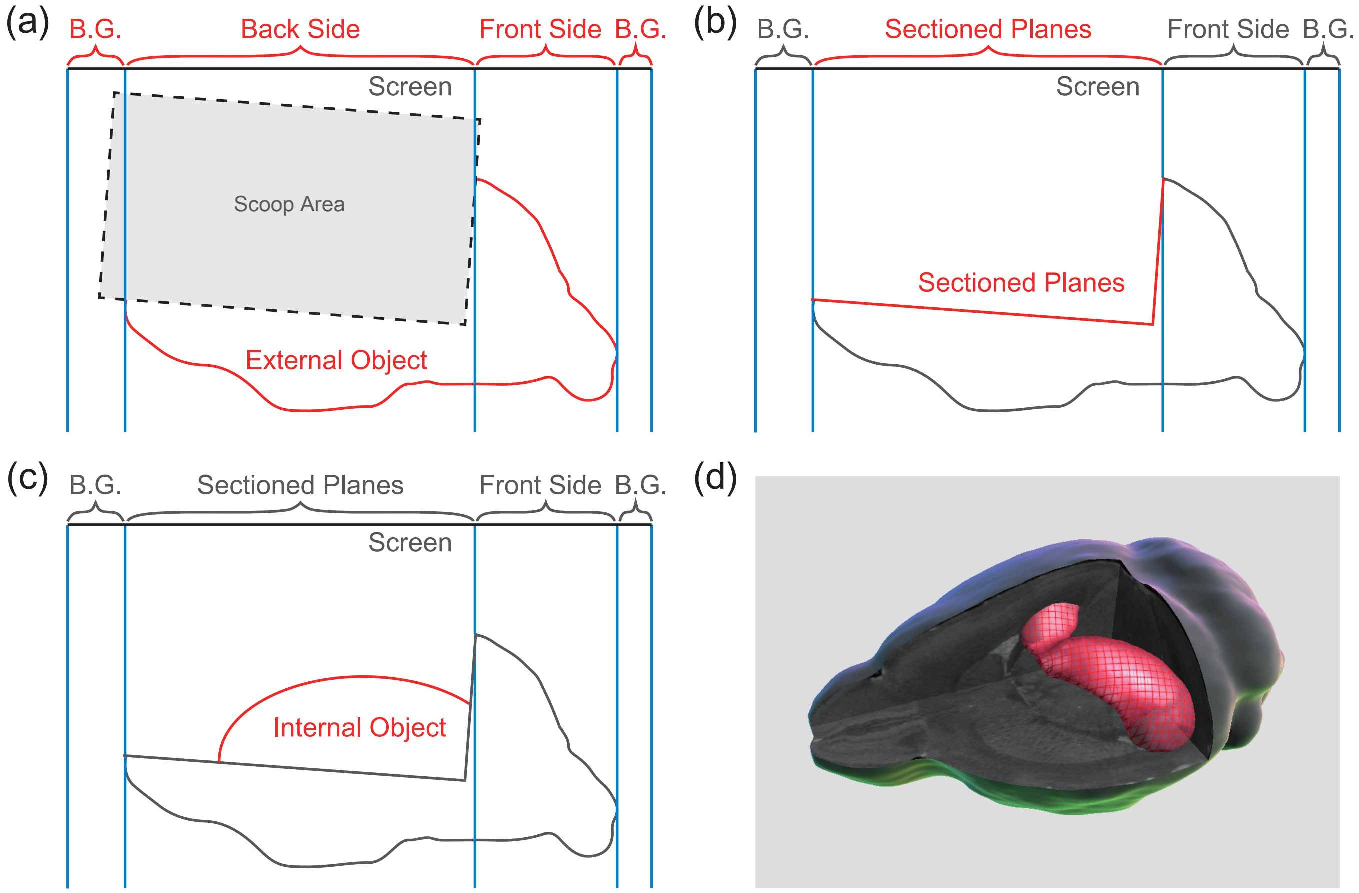

Supplementary Figure S2. Outline of the visualization method (a) First, render the external object and clip the scoop area. From the result of rendering, there are three states on the pixels on the screen: the front side is drawn, the back side is drawn, and the background is drawn. (b) Next, render and overwrite the sectioned planes of the scoop area to the pixel on which the back side of the screen is drawn. The texture of the sectioned planes are dynamically generated from the volume image each time. (c) Finally, render the internal object. Our method utilizes general depth buffering for the management of depth coordinates in 3D graphics. (d) An example of rendering results.
